# Histologic heterogeneity predicts patient prognosis of HER2‐positive metastatic breast cancer: A retrospective study based on SEER database

**DOI:** 10.1002/cam4.6469

**Published:** 2023-08-21

**Authors:** Yajie Wang, Yiran Liang, Fangzhou Ye, Dan Luo, Yuhan Jin, Yaming Li, Wenjing Zhao, Bing Chen, Lijuan Wang, Qifeng Yang

**Affiliations:** ^1^ Department of Breast Surgery, General Surgery Qilu Hospital of Shandong University Jinan China; ^2^ Pathology Tissue Bank Qilu Hospital of Shandong University Jinan China; ^3^ Research Institute of Breast Cancer Shandong University Jinan China

**Keywords:** HER2‐positive breast cancer, histologic heterogeneity, preferential distant metastasis, prognosis, SEER database

## Abstract

**Background:**

Human epidermal growth factor receptor 2‐positive (HER2+) metastatic breast cancer (MBC) is a subtype of breast cancer with a worse prognosis. Little is known about the relationship between histology and prognosis among different distant metastasis sites (DMS). Our aims were to explore the prognostic value of histologic subtypes in different DMS and screen out specific subtypes with particular DMS that need more attention in HER2+ MBC.

**Methods:**

HER2+ MBC patient data were obtained from the Surveillance, Epidemiology, and End Results (SEER) database between 2010 and 2014. Chi‐squared tests were utilized to compare histologic subtypes in four DMS. The logistic regression analyses were used to control confounding factors. The log‐rank tests were used to analyze the correlation of histologic subtype with disease‐specific survival and overall survival. The survival data was analyzed using Kaplan–Meier methods.

**Results:**

A total of 1174 HER2+ MBC patients were involved. First, the distribution of histological subtypes varied across metastatic sites, and the proportions of metastatic sites in different histological subtypes were also different. Furthermore, different histological subtypes within specific DMS showed divergent prognoses, and the different outcomes were shown by distinct DMS for specific histological subtypes. Among them, lobular carcinoma (ILC) subtypes showed the worst prognosis in bone metastasis, and lung metastasis predicted the worst prognosis in infiltration duct and lobular carcinoma (IDC‐ILC) subtypes. After further consideration of hormone receptor (HR) status, the IDC‐ILC subtype with liver metastasis in HR+/HER2+ MBC patients and the ILC subtype with bone metastasis in HR−/HER2+ MBC patients proved to be noteworthy.

**Conclusions:**

Histological subtypes are involved in determining the heterogeneity of HER2+ MBC patient prognosis, which is helpful to guide the prognosis prediction and monitoring of HER2+ breast cancer patients in clinics.

## 1 INTRODUCTION

Breast cancer is the most prevalent cancer and the second leading cause of cancer‐related deaths among women around the world,^1^ whose lifetime risk is one in eight individuals (approximately 13%), with 13.6 deaths per 100,000.^2^ Breast cancer is a heterogeneous disease, comprising numerous distinct characters in not only biological features but also clinical behaviors.^3^ According to the expression of estrogen receptor (ER), progesterone receptor (PR), and the amplification of human epidermal growth factor receptor 2 (HER2), breast cancer is recognized as four molecular subtypes: luminal A (ER‐positive, PR‐positive, HER2 negative); luminal B (ER‐positive, PR‐positive, HER2 negative or positive); HER2 type (HER2 positive, particularly aggressive); basal‐like or triple‐negative breast cancer (ER negative, PR negative, HER2 negative, TNBC),^4^ all of which are routinely used to stratify patients for prognostic prediction and treatment selection in the clinic, as well as to select patients for clinical trials and clinical database analysis.

HER2 is a tyrosine kinase receptor encoded by the gene ERBB2 (erythroblastic oncogene B). HER2+ breast cancer accounts for approximately 15%–20% of breast cancer, which is defined as high expression measured by immunohistochemistry status and/or amplification of HER2 detected by fluorescence in‐situ hybridization (FISH).^5^ Several anti‐HER2 agents are targeting the HER2 family both intracellularly and extracellularly as an adjuvant treatment before or after surgery, including trastuzumab, pertuzumab, trastuzumab emtansine (T‐DM1), lapatinib, and neratinib.^6^ However, it's reported that compared with HR‐positive and HER2‐negative subtypes, HER2+ breast cancer has a worse survival outcome because of its aggressive behavior and high recurrence rate.^7^ Reportedly, the median overall survival (OS) of early HER2+ breast cancer patients is currently 5 years,^8^ and distant metastasis is one of the most significant causes of poor prognosis. Despite advances in the prevention, detection, and adjuvant therapy of breast cancer,^9^ about 15%–24% of patients will develop metastatic disease after completing curative‐intent therapy, and 3%–10% present with de novo metastasis.^10^, ^11^ Once metastasis occurred, the survival time would be greatly shortened and the quality of life (QOL) would be seriously impaired, making metastasis the main cause of breast cancer‐related death.^12^

Predominately, distant metastatic organs of breast cancer include bone, lung, liver, and brain. Breast cancer exhibits a metastatic propensity to distinct organs, which is defined as metastatic heterogeneity and leads to varied responses to treatment and patient prognosis.^13^ The essence of metastasis heterogeneity is the diversity of primary tumors in terms of intrinsic molecular expression and clinicopathological features. At the molecular level, several key molecules are responsible for metastatic heterogeneity in breast cancer. For instance, the FUS/circEZH2/KLF5 feedback loop enhanced epithelial‐mesenchymal transition, contributing to CXCR4‐induced liver metastasis.^14^ On the other hand, clinicopathological features also play an essential role in modulating metastatic heterogeneity. A retrospective study analyzing clinical records of 2193 breast cancer patients in Caro Oncology Center database, revealed that age less than 60 years, larger tumors, LN positivity, high tumor grade, and HER2 positivity were associated with a higher incidence of brain metastasis.^15^ Another study reported that young age, invasive ductal carcinoma, higher pathological grade, and subtype of TNBC and HER2+ breast cancer were risk factors for liver metastasis.^16^ Usually, the expression level of all key molecules cannot be exhaustively monitored, but clinical features such as histologic subtypes are clinically available. Whereas, to the best of our knowledge, there has not been a population‐based evaluation of the impact of histological heterogeneity on prognosis prediction in HER2+ breast cancer at different metastatic sites.

In this study, we first analyzed the proportion of four metastatic sites and the prognosis of different metastatic sites in HER2+ MBC patients. We then compared the proportions of histologic subtypes at different metastatic sites and metastatic sites in different histologic subtypes. And then, we assessed the prognostic divergences in histologic subtypes within specific DMS and in specific subtype within distinct DMS. And specific subtypes with particular DMS that need to be paid more attention were screened out. Finally, the role of HR expression status in determining the prognostic heterogeneity of patients with distant metastasis by histological subtype was further discussed. This study helps us fully understand the impact of histologic heterogeneity on patient prognosis heterogeneity, and helps to guide prognosis monitoring of HER2+ breast cancer patients in clinical practice.

## 2 METHODS

### 2.1 Data source

Data on patients with HER2+ metastatic breast cancer (MBC) were extracted from the Surveillance, Epidemiology, and End Results (SEER) database (http://seer.cancer.gov/), which is a population‐based database that collects data from 18 cancer registries in the United States since 2010. For this investigation, the information of each patient was collected, including age at diagnosis, marital status, grade, tumor laterality, histologic subtype, tumor size, node stage, radiotherapy status, chemotherapy status, and survival data. The histological types of tumors are classified according to the International Classification of Diseases for Oncology (ICD‐O), 3rd edition. Tumor stage is performed according to the American Joint Committee on Cancer (AJCC) staging system, 7th edition.

### 2.2 Patient selection

We download the data of breast cancer patients diagnosed over a 5‐year period between 2010 and 2014. The major inclusion criteria are listed as follows: female, 18–85 years of age at diagnosis, 2010–2014 diagnosed, breast cancer as the first and only malignant cancer diagnosis, HER2+, available complete dates, active follow‐up (having a known cause of death and survival time), histologic types. We excluded patients who lacked a histologically confirmed diagnosis, identified by death certificate or autopsy, and those without metastasis and with multiple distant metastatic sites. Besides, we also excluded older breast cancer patients over 85 at diagnosis to minimized the impact of multiple comorbidities as well as non‐receipt of guideline‐concordant care due to comorbidity or life expectancy.^17^, ^18^

The five most prevalent and well‐defined histologic subtypes were included in this analysis: infiltrating duct carcinoma (IDC, ICD‐O‐38500/3), lobular carcinoma (ILC, ICD‐O‐38520/3), infiltration duct and lobular carcinoma (IDC‐ILC, ICD‐O‐38522/3), infiltrating duct mixed with other type (IDC‐oth, ICD‐O‐38523/3), and inflammatory carcinoma (IBC, ICD‐O‐38530/3).

### 2.3 Statistical analysis

The differences in histologic subtype distribution between the four metastases groups were analyzed using Pearson's chi‐squared test. In order to control confounding factors, we conducted univariate logistic regression analysis. All factors that were statistically significant in univariate analysis were entered into the multivariable logistic regression analysis. Log‐rank tests were utilized to screen out specific histologic subtype of four DMS in prognosis prediction. Disease‐specific survival (DSS) was defined as the interval from the date of diagnosis to the date of breast cancer‐related death, while OS was defined as the interval from the date of diagnosis to the death caused by any reasons or the last follow‐up, calculating by hazard ratios (HRs) with 95% confidence intervals (CIs). The survival data of each metastasis group or histologic subtype was analyzed using Kaplan–Meier methods. Statistical analyses were performed using SPSS version 22. All tests were two‐sided, and the value of *p* < 0.05 was considered statistically significant.

## 3 RESULTS

### 3.1 Patient characteristics

**FIGURE 1:**
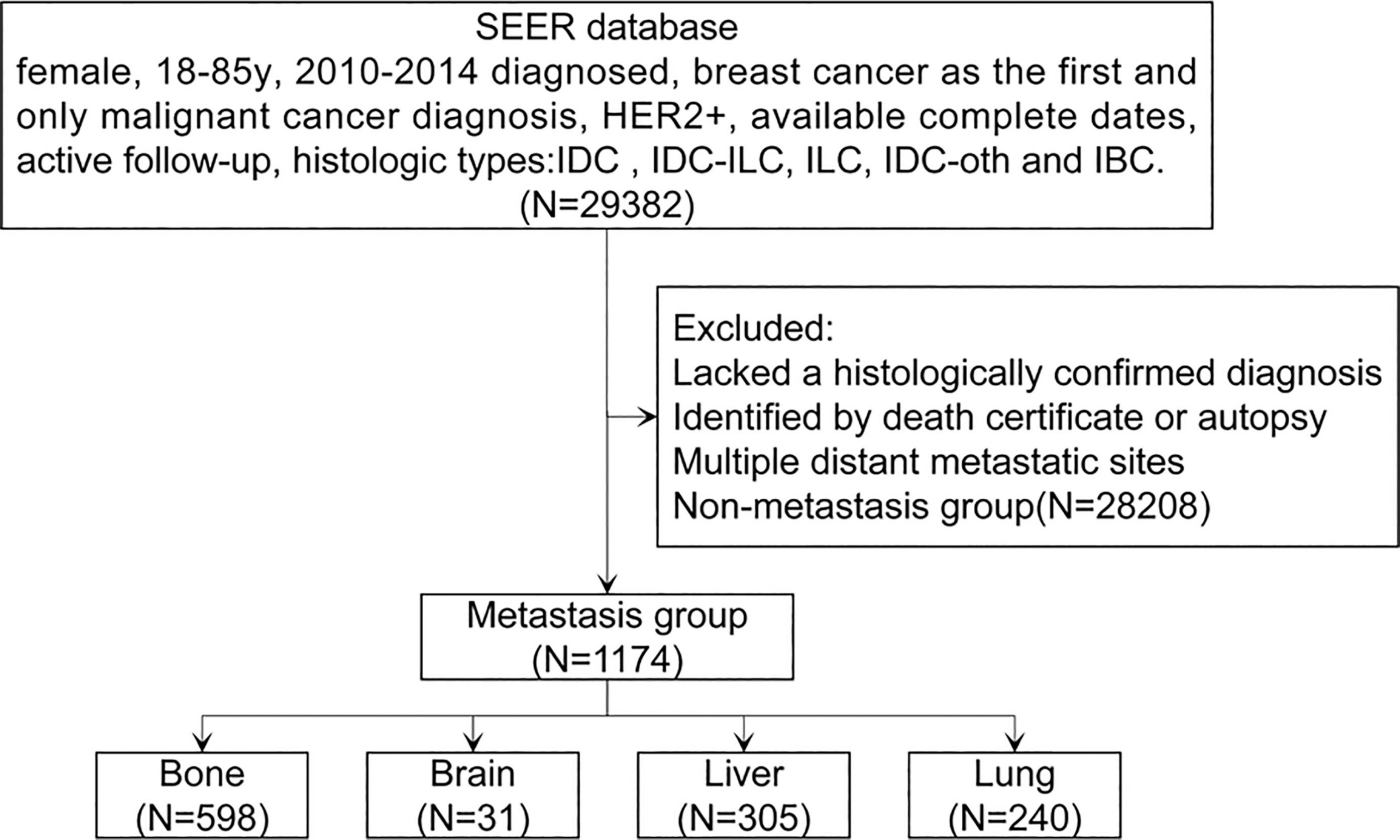
Flow chart of the screened patients. HER2, human epidermal growth factor receptor 2; IBC, inflammatory carcinoma; IDC, infiltrating duct carcinoma; IDC‐ILC, infiltration duct and lobular carcinoma; ILC, lobular carcinoma; IDC‐oth, infiltrating duct mixed with other type; SEER, Surveillance, Epidemiology, and End Result.

A total of 1174 HER2+ MBC patients entered the study. The flow chart of the inclusion criteria and the reasons for exclusion are presented in Figure 1. According to metastasis status, the population demographics, clinicopathological features, and treatment characteristics of patients with HER2+ MBC were presented in Table 1. The proportions of metastatic sites and histological subtypes in all patients were shown in Figure S1A. We then analyzed both DSS and OS stratified by metastatic sites in HER2+ MBC patients included in this study, demonstrating that patients with different metastatic sites have different prognoses (*p* < 0.0001). Concretely, bone metastasis showed a relatively favorable prognosis, while brain metastasis had the worst prognosis among all groups (Figure S1B).

**TABLE 1:** Demographic and baseline characteristics of patients with different metastasis patterns of HER2+ MBC.

Characteristics	Bone only (50.9) *N* = 598 (%)	Brain only (2.6) *N* = 31 (%)	Liver only (26.0) *N* = 305 (%)	Lung only (20.4) *N* = 240 (%)	Total single metastasis sites *N* = 1174 (%)
Patient age, years					
<50	195 (32.6)	9 (29.0)	113 (37.1)	53 (22.1)	370 (31.5)
50–75	367 (61.4)	21 (67.7)	175 (57.4)	156 (65.0)	719 (61.2)
>75	36 (6.0)	1 (3.2)	17 (5.6)	31 (12.9)	85 (7.2)
Race					
White	442 (73.9)	20 (64.5)	228 (74.8)	182 (75.8)	872 (74.3)
Black	107 (17.9)	8 (25.8)	53 (17.4)	42 (17.5)	210 (17.9)
Other^a^	45 (7.5)	3 (9.7)	24 (7.9)	16 (6.7)	88 (7.5)
Unknown	4 (0.7)	0	0	0	4 (0.3)
Marital status					
Unmarried^b^	280 (46.8)	18 (58.1)	134 (43.9)	121 (50.4)	553 (47.1)
Married	280 (46.8)	12 (38.7)	155 (50.8)	103 (42.9)	550 (46.9)
Unknown	38 (6.4)	1 (3.2)	16 (5.3)	16 (6.7)	71 (6.1)
Grade					
Well/Moderately; Grade I/II	242 (40.5)	5 (16.1)	96 (31.5)	53 (22.1)	396 (33.7)
Poorly/Undifferentiated; Grade III/IV	280 (46.8)	19 (61.3)	180 (59.0)	168 (70.0)	647 (55.1)
Unknown	76 (12.7)	7 (22.6)	29 (9.5)	19 (7.9)	131 (11.2)
Laterality					
Left	321 (53.7)	17 (54.8)	151 (49.5)	107 (44.6)	596 (50.8)
Right	270 (45.2)	14 (45.2)	153 (50.2)	131 (54.6)	568 (48.4)
Bilateral	7 (1.2)	0	1 (0.3)	2 (0.8)	10 (0.9)
Unknown	0	0	0	0	0
Histologic subtype					
IDC	505 (84.5)	26 (83.9)	279 (91.5)	221 (92.1)	1031 (87.8)
ILC	36 (6.0)	1 (3.2)	6 (2.0)	1 (0.4)	44 (3.8)
IDC‐ILC	29 (4.9)	2 (6.5)	13 (4.3)	6 (2.5)	50 (4.3)
IDC‐oth	13 (2.2)	0	3 (1.0)	1 (0.4)	17 (1.5)
IBC	15 (2.5)	2 (6.5)	4 (1.3)	11 (4.6)	32 (2.7)
Tumor size (mm)					
≤50	267 (44.7)	14 (45.2)	150 (49.2)	95 (39.6)	526 (44.8)
>50	274 (45.8)	13 (41.9)	125 (41.0)	136 (56.7)	548 (46.7)
Unknown	57 (9.5)	4 (12.9)	30 (9.8)	9 (3.8)	100 (8.5)
Node stage					
Negative	124 (20.7)	5 (16.1)	63 (20.7)	38 (15.8)	230 (19.6)
Positive	446 (74.6)	24 (77.4)	227 (74.4)	195 (81.3)	892 (76.0)
Unknown	28 (4.7)	2 (6.5)	15 (4.9)	7 (2.9)	52 (4.4)
Radiotherapy status					
No/Unknown	443 (74.1)	16 (51.6)	253 (83.0)	217 (90.42)	929 (79.1)
Yes	155 (25.9)	15 (48.4)	52 (17.1)	23 (9.58)	245 (20.9)
Chemotherapy status					
No/Unknown	121 (20.2)	9 (29.0)	57 (18.7)	54 (22.5)	241 (20.5)
Yes	477 (79.8)	22 (71.0)	248 (81.3)	186 (77.5)	933 (79.5)

Abbreviations: IBC, inflammatory carcinoma; IDC, infiltrating duct carcinoma; ILC, lobular carcinoma; MBC, metastatic breast cancer.

^a^ Other includes American Indian/Alaskan native, Asian/Pacific Islander, and others—unspecified.

^b^ Unmarried means divorced, separated, single (never married), unmarried, domestic partner, and widowed.

### 3.2 Distribution of metastatic sites in specific histologic subtype and histologic subtypes in specific metastatic site

**FIGURE 2:**
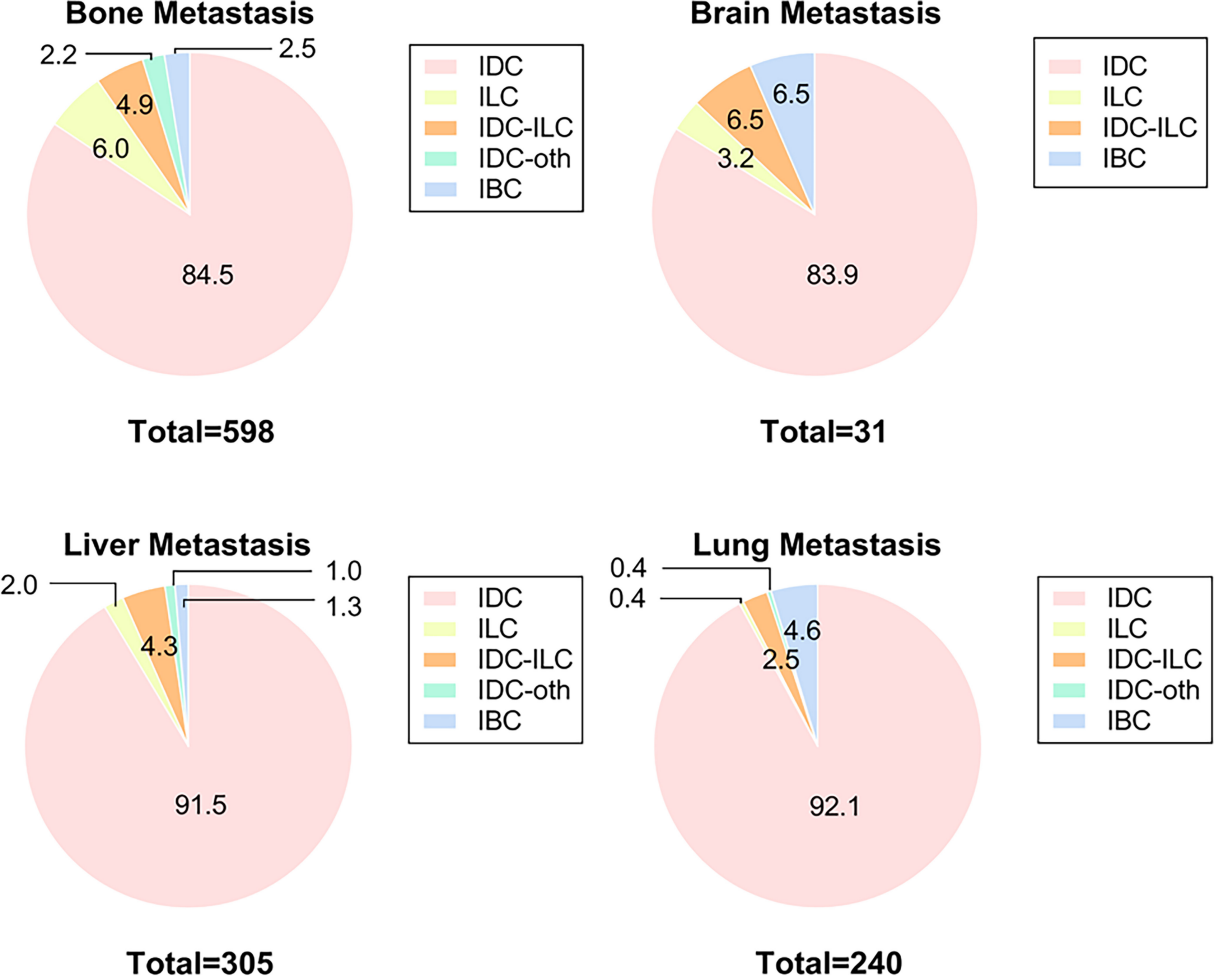
Pie graphs that the proportion of histologic subtype in each distant metastatic site. IBC, inflammatory carcinoma; IDC, infiltrating duct carcinoma; IDC‐ILC, infiltration duct and lobular carcinoma; IDC‐oth, infiltrating duct mixed with other type; ILC, lobular carcinoma.

**FIGURE 3:**
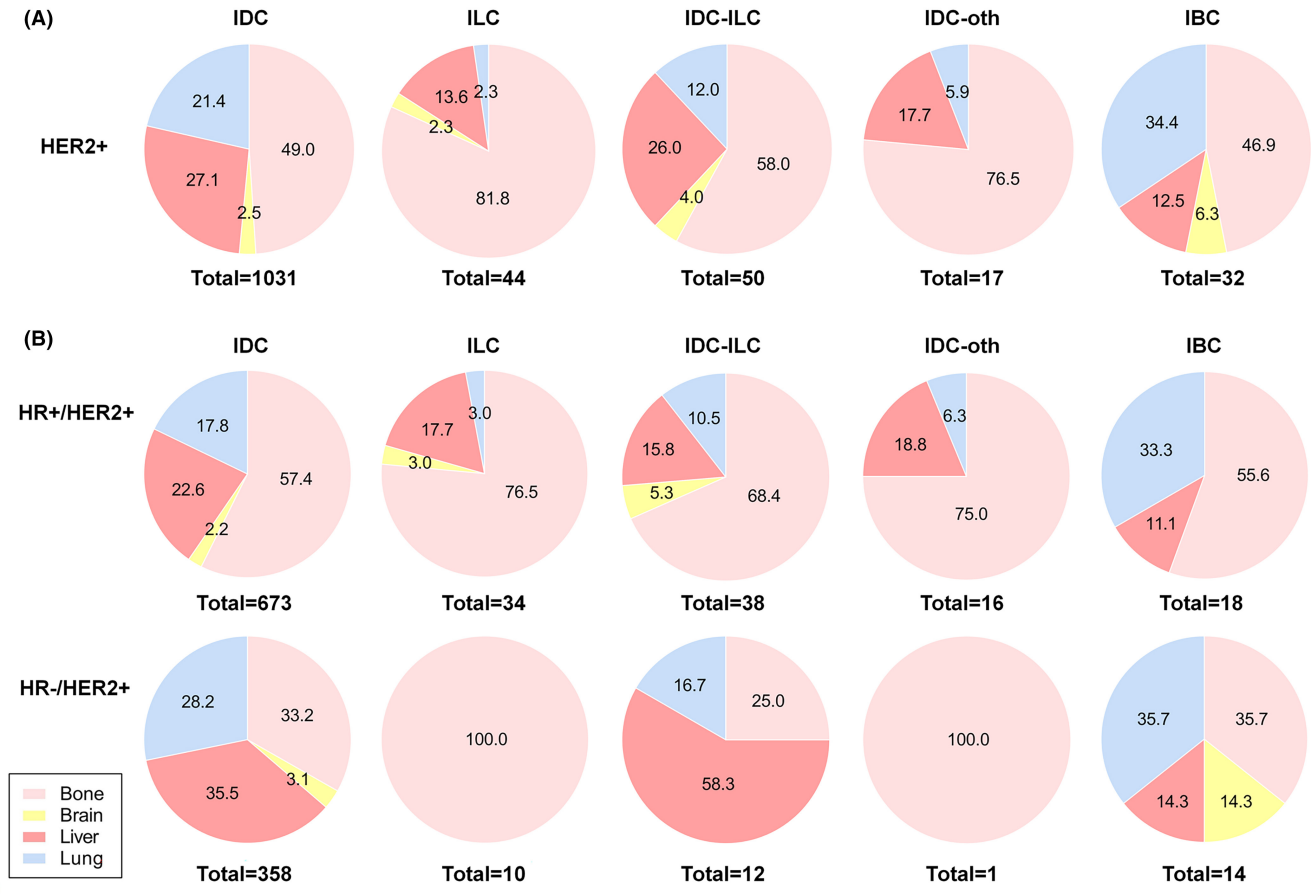
Pie graphs that the proportion of distant metastatic sites in each histologic subtype. (A) Pie graphs that the proportion of distant metastatic sites in each histologic subtype in HER2+ MBC. (B) Pie graphs that the proportion of distant metastatic sites in each histologic subtype in HR+/HER2+ MBC and HR−/HER2+ MBC. HER2, human epidermal growth factor receptor 2; HR, hazard ratio; IBC, inflammatory carcinoma; IDC, infiltrating duct carcinoma; ILC, lobular carcinoma; IDC‐ILC, infiltration duct and lobular carcinoma; IDC‐oth, infiltrating duct mixed with other type; MBC, metastatic breast cancer.

To explore whether the significant difference in the prognosis of different metastatic sites is related to histological heterogeneity. We first analyzed the distribution of histologic subtypes in four metastatic sites. The results showed that there were statistical differences (*p* = 0.001) in the distribution of histological subtypes among different metastatic sites (Table S1). And the detailed information was illustrated in the pie chart of Figure 2. We then compared the proportion of metastatic sites in different histological subtypes. The detailed information was exhibited in the pie chart of Figure 3A.

### 3.3 Impact of histologic heterogeneity on survival of HER2‐positive MBC

**FIGURE 4:**
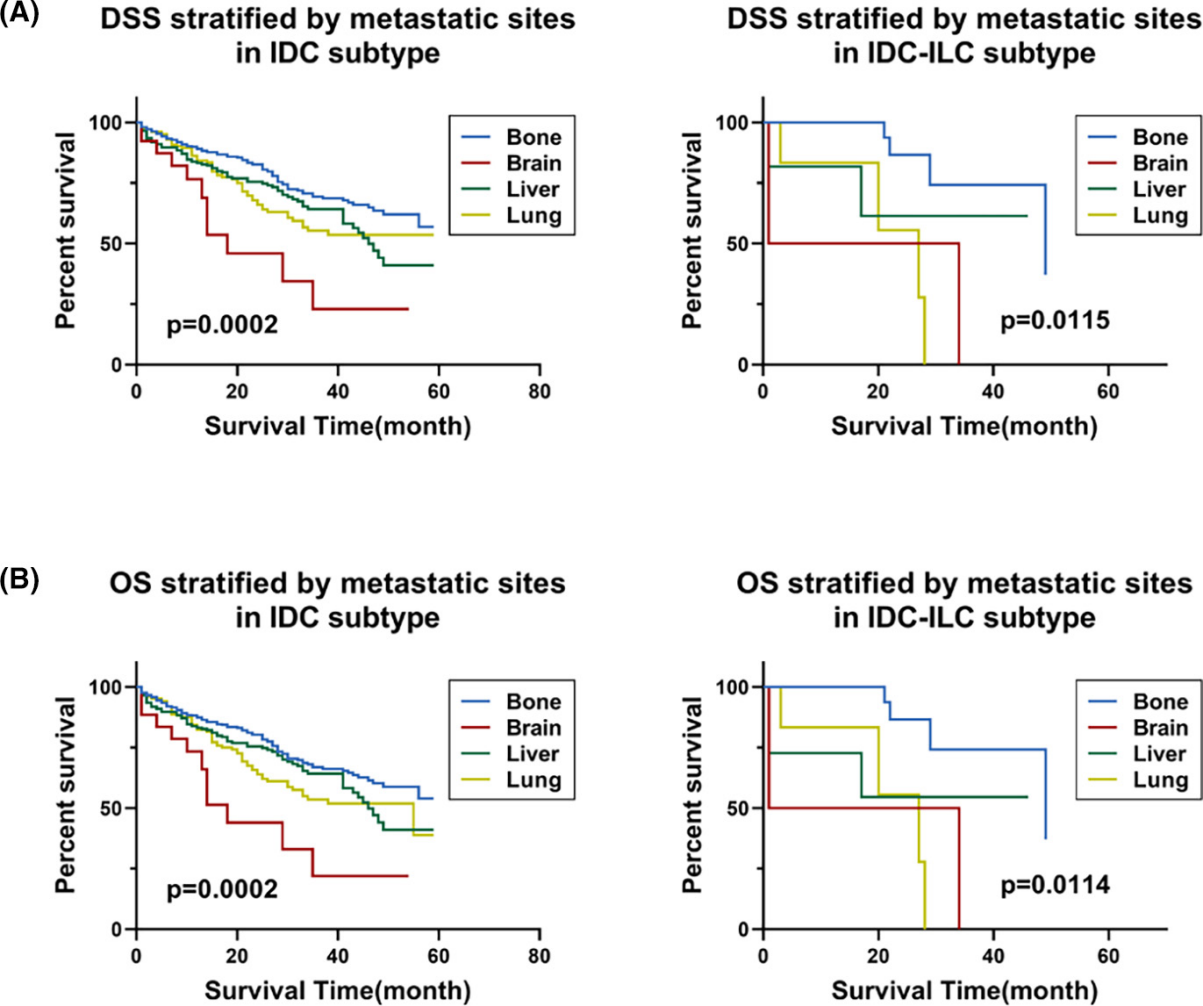
Kaplan–Meier survival curve of DSS and OS stratified by metastatic sites in IDC and IDC‐ILC subtypes. (A) DSS stratified by metastatic sites in IDC and IDC‐ILC subtypes. (B) OS stratified by metastatic sites in IDC and IDC‐ILC subtypes. DSS, disease‐specific survival; IDC, infiltrating duct carcinoma; IDC‐ILC, infiltration duct and lobular carcinoma; OS, overall survival.

After adjusting confounding factors, histological subtype was proved to be one of the independent indicators for DMS (Tables S2 and S3). We then evaluated whether histologic subtypes among common distant metastatic sites could affect the outcomes of HER2+ breast cancer patients. Overall, there was no statistical difference in the prognosis of histological subtypes in four metastatic sites (Figure S2). The results of log‐rank tests demonstrated that compared with IDC, bone metastasis in patients with ILC had significantly worse DSS (HR = 2.044) (Table 2). Regarding OS, the same results were found in ILC subtype of bone metastasis (HR = 1.808) (Table 3). We also evaluated the prognostic differences among the four DMS of each histologic subtype. As shown in Figure 4, the differences between four metastatic sites of IDC subtype and IDC‐ILC subtypes were statistically significant, both in DSS and in OS. Furthermore, for DSS, compared with bone metastasis, the most striking hazard ratio was observed in brain metastasis in total MBC patients (HR = 2.933), followed by lung metastasis (HR = 1.616) and liver metastasis (HR = 1.557). In IDC group, liver metastasis (HR = 1.670) had a more pronounced hazard ratio than lung metastasis (HR = 1.636). In addition, for patients with IDC‐ILC, lung metastasis indicated the worst prognosis (HR = 4.780) (Table 4). Considering OS, similar results were obtained for IDC group and total group. Besides, the same results of IDC‐ILC subtype (HR = 4.666) were found in lung metastasis (Table 5).

**TABLE 2:** Log‐rank analysis of DSS for specific DMS with different histologic subtypes.

Histologic subtype	Bone metastasis		Brain metastasis		Liver metastasis		Lung metastasis	
	HR (95% CI)	*p*	HR (95% CI)	*p*	HR (95% CI)	*p*	HR (95% CI)	*p*
HER2+								
IDC	REF	REF	REF	REF	REF	REF	REF	REF
ILC	2.044 (1.169–3.575)*	**0.012**	‐	‐	0.631 (0.088–4.537)	0.648	3.784 (0.520–27.546)	0.189
IDC‐ILC	1.171 (0.514–2.668)	0.707	1.787 (0.376–8.496)	0.466	1.094 (0.346–3.465)	0.878	2.236 (0.811–6.164)	0.120
IDC‐oth	0.000 (0.000–1.810E+137)	0.947	‐	‐	0.000 (0.000–5.130E+176)	0.959	1.393 (0.191–10.144)	0.744
IBC	0.956 (0.303–3.015)	0.939	1.309 (0.164–10.465)	0.800	0.570 (0.127–2.567)	0.568	0.744 (0.234–2.368)	0.617
HR+/HER2+								
IDC	REF	REF	REF	REF	REF	REF	REF	REF
ILC	1.575 (0.791–3.137)	0.196	‐	‐	0.654 (0.090–4.748)	0.675	6.559 (0.859–50.056)	0.070
IDC‐ILC	1.170 (0.474–2.891)	0.733	2.080 (0.381–11.340)	0.398	2.859 (0.876–9.338)	0.082	3.013 (0.899–10.103)	0.074
IDC‐oth	‐	‐	‐	‐	‐	‐	1.821 (0.243–13.667)	0.560
IBC	0.418 (0.058–3.003)	0.386	‐	‐	0.898 (0.123–6.551)	0.916	1.137 (0.270–4.797)	0.861
HR−/HER2+								
IDC	REF	REF	REF	REF	REF	REF	REF	REF
ILC	3.751 (1.401–10.042)**	**0.009**	‐	‐	‐	‐	‐	‐
IDC‐ILC	1.476 (0.197–11.068)	0.705	‐	‐	0.045 (0.000–46.195)	0.381	2.607 (0.352–19.314)	0.348
IDC‐oth	‐	‐	‐	‐	‐	‐	‐	‐
IBC	2.651 (0.621–11.318)	0.188	1.045 (0.116–9.401)	0.968	0.046 (0.000–6545.936)	0.610	0.508 (0.070–3.702)	0.508

Abbreviations: DMS, distant metastasis sites; DSS, disease‐specific survival; IBC, inflammatory carcinoma; IDC, infiltrating duct carcinoma; ILC, lobular carcinoma; IDC‐ILC, infiltration duct and lobular carcinoma; IDC‐oth, infiltrating duct mixed with other type.

Bold values indicates *p<0.05; **p<0.01; ***p<0.001.

**TABLE 3:** Log‐rank analysis of OS for specific DMS with different histologic subtypes.

Histologic subtype	Bone metastasis		Brain metastasis		Liver metastasis		Lung metastasis	
	HR (95% CI)	*p*	HR (95% CI)	*p*	HR (95% CI)	*p*	HR (95% CI)	*p*
HER2+								
IDC	REF	REF	REF	REF	REF	REF	REF	REF
ILC	1.808 (1.038–3.149)*	**0.037**	‐	‐	0.600 (0.084–4.308)	0.611	3.344 (0.474–25.002)	0.222
IDC‐ILC	1.043 (0.459–2.369)	0.920	1.660 (0.354–7.778)	0.520	1.382 (0.507–3.767)	0.528	2.062 (0.750–5.668)	0.161
IDC‐oth	0.000 (0.000–8.041E+128)	0.944	‐	‐	0.000 (0.000–2.272E+173)	0.958	1.330 (0.183–9.669)	0.778
IBC	0.854 (0.271–2.689)	0.788	1.190 (0.151–9.401)	0.895	0.494 (0.069–3.555)	0.484	0.927 (0.338–2.539)	0.882
HR+/HER2+								
IDC	REF	REF	REF	REF	REF	REF	REF	REF
ILC	1.385 (0.698–2.746)	0.352	‐	‐	0.654 (0.090–4.748)	0.675	6.027 (0.793–45.800)	0.083
IDC‐ILC	1.029 (0.418–2.535)	0.960	1.792 (0.344–9.326)	0.488	2.859 (0.876–9.338)	0.082	2.849 (0.852–9.521)	0.089
IDC‐oth	‐	‐	‐	‐	‐	‐	1.768 (0.236–13.241)	0.579
IBC	0.372 (0.052–2.673)	0.326	‐	‐	0.898 (0.123–6.551)	0.916	1.090 (0.259–4.591)	0.906
HR−/HER2+								
IDC	REF	REF	REF	REF	REF	REF	REF	REF
ILC	3.359 (1.266–8.910)*	**0.015**	‐	‐	‐	‐	‐	‐
IDC‐ILC	1.386 (0.186–10.350)	0.750	‐	‐	0.516 (0.071–3.759)	0.514	2.259 (0.307–16.643)	0.424
IDC‐oth	‐	‐	‐	‐	‐	‐	‐	‐
IBC	2.413 (0.568–10.245)	0.233	1.045 (0.116–9.401)	0.968	0.000 (0.000–6.524E+256)	0.971	0.928 (0.225–3.839)	0.918

Abbreviations: DMS, distant metastasis sites; IBC, inflammatory carcinoma; IDC, infiltrating duct carcinoma; ILC, lobular carcinoma; IDC‐ILC, infiltration duct and lobular carcinoma; IDC‐oth, infiltrating duct mixed with other type; OS, overall survival.

Bold values indicates *p<0.05; **p<0.01; ***p<0.001.

**TABLE 4:** Log‐rank analysis of DSS for specific histologic subtypes with different DMS.

Histologic subtype	Bone metastasis		Brain metastasis		Liver metastasis		Lung metastasis	
	HR (95% CI)	*p*	HR (95% CI)	*p*	HR (95% CI)	*p*	HR (95% CI)	*p*
HER2+								
IDC	REF	REF	3.058 (1.641–5.698)***	**<0.001**	1.670 (1.259–2.215)***	**<0.001**	1.636 (1.200–2.229)**	**0.002**
ILC	REF	REF	‐	‐	0.501 (0.065–3.838)	0.506	3.500 (0.439–27.908)	0.237
IDC‐ILC	REF	REF	4.176 (0.792–22.013)	0.092	1.982 (0.469–8.384)	0.352	4.780 (1.236–18.482)*	**0.023**
IDC‐oth	REF	REF	‐	‐	0.015 (0.610–147346.844)	0.610	‐	‐
IBC	REF	REF	4.984 (0.443–56.031)	0.193	0.916 (0.095–8.858)	0.940	1.319 (0.265–6.554)	0.735
Total	REF	REF	2.933 (1.688–5.096)***	**<0.001**	1.557 (1.193–2.033)**	**0.001**	1.616 (1.214–2.153)**	**0.001**
HR+/HER2+								
IDC	REF	REF	2.703 (1.179–6.197)*	**0.019**	1.448 (1.010–2.077)*	**0.044**	1.089 (0.701–1.691)	0.705
ILC	REF	REF	‐	‐	0.631 (0.079–5.052)	0.664	4.858 (0.555–42.500)	0.153
IDC‐ILC	REF	REF	2.923 (0.540–15.836)	0.213	5.816 (1.224–27.649)*	**0.027**	3.906 (0.880–17.344)	0.073
IDC‐oth	REF	REF	‐	‐	0.015 (0.610–147346.844)	0.610	‐	‐
IBC	REF	REF	‐	‐	2.834 (0.174–46.154)	0.464	3.381 (0.306–37.358)	0.320
Total	REF	REF	2.659 (1.293–5.469)**	**0.008**	1.448 (1.032–2.031)*	**0.032**	1.219 (0.822–1.809)	0.325
HR−/HER2+								
IDC	REF	REF	3.841 (1.450–10.175)**	**0.007**	2.105 (1.258–3.522)**	**0.005**	2.627 (1.553–4.446)***	**<0.001**
ILC	REF	REF	‐	‐	‐	‐	‐	‐
IDC‐ILC	REF	REF	‐	‐	1.000 (0.000–1.464E+18)	>0.999	270.764 (0.000–3.385E+16)	0.735
IDC‐oth	REF	REF	‐	‐	‐	‐	‐	‐
IBC	REF	REF	1.490 (0.120–18.516)	0.757	‐	‐	0.456 (0.041–5.103)	0.524
Total	REF	REF	3.222 (1.337–7.768)**	**0.009**	1.660 (1.040–2.649)*	**0.034**	2.146 (1.335–3.447)**	**0.002**

Abbreviations: DMS, distant metastasis sites; DSS, disease‐specific survival; IBC, inflammatory carcinoma; IDC, infiltrating duct carcinoma; ILC, lobular carcinoma; IDC‐ILC, infiltration duct and lobular carcinoma; IDC‐oth, infiltrating duct mixed with other type.

Bold values indicates *p<0.05; **p<0.01; ***p<0.001.

**TABLE 5:** Log‐rank analysis of OS for specific DMS with different histologic subtypes.

Histologic subtype	Bone metastasis		Brain metastasis		Liver metastasis		Lung metastasis	
	HR (95% CI)	*p*	HR (95% CI)	*p*	HR (95% CI)	*p*	HR (95% CI)	*p*
HER2+								
IDC	REF	REF	2.944 (1.624–5.339)***	**<0.001**	1.552 (1.183–2.038)**	**0.002**	1.586 (1.181–2.129)**	**0.002**
ILC	REF	REF		‐	0.501 (0.065–3.838)	0.506	3.500 (0.439–27.908)	0.237
IDC‐ILC	REF	REF	4.219 (0.802–22.197)	0.089	2.587 (0.687–9.739)	0.160	4.666 (1.212–17.923)*	**0.025**
IDC‐oth	REF	REF	‐	‐	0.015 (0.610–147346.844)	0.610	‐	‐
IBC	REF	REF	5.745 (0.503–65.625)	0.159	0.895 (0.093–8.642)	0.923	1.774 (0.396–7.946)	0.454
Total	REF	REF	2.838 (1.665–4.837)***	**<0.001**	1.488 (1.151–1.925)**	**0.002**	1.602 (1.219–2.106)**	**0.001**
HR+/HER2+								
IDC	REF	REF	2.790 (1.293–6.021)**	**0.009**	1.281 (0.900–1.823)	0.169	1.035 (0.69–1.578)	0.874
ILC	REF	REF	‐	‐	0.631 (0.079–5.052)	0.664	4.858 (0.555–42.500)	0.153
IDC‐ILC	REF	REF	2.923 (0.540–15.836)	0.213	5.816 (1.224–27.649)*	**0.027**	3.906 (0.880–17.344)	0.073
IDC‐oth	REF	REF	‐	‐	0.015 (0.610–147346.844)	0.610	‐	‐
IBC	REF	REF	‐	‐	2.834 (0.174–46.154)	0.464	3.381 (0.306–37.358)	0.320
Total	REF	REF	2.702 (1.368–5.335)**	**0.004**	1.301 (0.932–1.815)	0.121	1.161 (0.793–1.699)	0.442
HR−/HER2+								
IDC	REF	REF	3.422 (1.302–8.993)*	**0.013**	2.098 (1.282–3.433)**	**0.003**	2.620 (1.585–4.331)***	**<0.001**
ILC	REF	REF	‐	‐	‐	‐	‐	‐
IDC‐ILC	REF	REF	‐	‐	30023.851 (0.000–5.316E+209)	0.996	58140.936 (0.000–1.029E+210)	0.964
IDC‐oth	REF	REF	‐	‐	‐	‐	‐	‐
IBC	REF	REF	1.816 (0.141–23.414)	0.648	‐	‐	0.790 (0.108–5.764)	0.816
Total	REF	REF	2.943 (1.227–7.059)*	**0.016**	1.730 (1.105–2.708)*	**0.017**	2.230 (1.416–3.512)**	**0.001**

Abbreviations: DMS, distant metastasis sites; IBC, inflammatory carcinoma; IDC, infiltrating duct carcinoma; ILC, lobular carcinoma; IDC‐ILC, infiltration duct and lobular carcinoma; IDC‐oth, infiltrating duct mixed with other type; OS, overall survival.

Bold values indicates *p<0.05; **p<0.01; ***p<0.001.

### 3.4 Roles of HR status in histological subtypes affecting prognosis of HER2‐positive MBC

HER2+ breast cancer is divided into two subtypes: HR+/HER2+ and HR−/HER2+. As shown in Figure 3B, there was a considerable discrepancy in the proportions of distant metastatic sites by histologic subtypes between HR+/HER2+ and HR−/HER2+ MBC. Log‐rank analyses demonstrated that the prognostic differences between the five histologic subtypes in each DMS were influenced by HR status. Among HR+/HER2+ subgroups, there was no significant difference between subgroups. While in HR−/HER2+ subgroup, the results were approximately the same as the HER2+ group, showing that bone metastasis patients with ILC had a significantly worse DSS (HR = 3.751) than that of IDC patients (Table 2). In terms of OS, the results were the same as DSS (Table 3). Meanwhile, the prognostic differences between the four DMS for each histological subtype were related to HR status. In HR+/HER2+ subgroup, same as HER2+ group, IDC patients with brain (HR = 2.703), and liver (HR = 1.636) metastasis showed worse prognosis. However, unlike the HER2+ group, on the whole, there was no significant difference in the prognosis between lung metastasis and bone metastasis. In IDC‐ILC subgroup, patients with liver metastasis had a much worse DSS (HR = 5.816) than those with bone metastasis. In HR−/HER2+ subgroups, the results of subgroups were consistent with those of HER2+ subgroups, except that IDC‐ILC subtype with lung metastasis showed no difference compared with bone metastasis (Table 4). Considering OS, the results were found in accordance with DSS, except that IDC subtype with liver metastasis was not different from IDC subtype with bone metastasis (Table 5).

## 4 DISCUSSION

Breast cancer is a heterogeneous disease, which mainly classifies into luminal A, luminal B, HER2, and TNBC, based on histological and molecular features.^19^, ^20^ HER2+ breast cancer was a subtype with a poor prognosis mainly due to the aggressive behavior and metastatic potential, and 20%–30% of patients will relapse toward a metastatic disease.^21^ While HER2 inhibitors are currently available, none of them achieves a cure in metastatic settings.^22^ Significantly, previous studies have proved that the metastatic propensity to distant organs in breast cancer could lead to treatment response and prognosis divergence, which is associated with intrinsic clinicopathological features. For instance, breast cancer liver metastasis was reported to be associated with young age, IDC, higher tumor grade, and subtype of TNBC and HER2+ were risk factors for developing liver metastasis.^16^ Moreover, patients of younger age (<60 years old), white race, lower grade, lower T stage, not combining visceral metastasis tended to have a better outcome.^23^ Whereas, in HER2+ MBC, the clinicopathological factors influencing its prognosis divergence in four distant metastatic sites are poorly understood. In this study, we explored the impact of histologic heterogeneity on prognosis in HER2+ MBC and revealed the significant role of certain histologic subtypes with specific DMS in predicting the prognosis of patients.

Bone, brain, liver, and lung are the four preferential metastatic organs of breast cancer. It has been reported that HER2+ and TNBC subtypes exhibited a four times greater risk of brain metastasis than luminal A/B subtypes, while luminal A and B tumors preferentially colonized bone metastasis rather than brain.^24^, ^25^ So, we first analyzed the proportion of four metastatic sites in HER2+ MBC patients. As a whole, bone metastasis was the most common metastatic site, which is the same as distant metastasis organ distribution of overall MBC.^26^ Though there was a high incidence of brain metastases among patients with HER2+ MBC and triple‐negative MBC,^27^ the proportion of brain metastasis was still the least. The prognosis of MBC depends on both hormonal receptor status and the first site of metastases, with a better prognosis for positive HR status, with the best prognosis for bone metastasis and the worst for brain metastases.^28^, ^29^, ^30^ Thus, we then compared the differences of both DSS and OS between four distant metastases in overall HER2+ MBC groups. Similarly, our results showed that bone metastasis portended a more favorable prognosis and brain metastasis had the worst prognosis.

Previous studies have proved that patients with different prognoses have divergences in clinicopathological features, suggesting that our observation of clinicopathological features may be useful in predicting the outcomes of patients. Wang et al. analyzed the clinicopathological features and prognosis of patients with different metastatic sites in stage IV breast cancer and reported that age, race, marital status, grade, tumor subtype, tumor size, surgery, and treatment history were independent prognostic factors of stage IV breast cancer patients.^31^ Besides, relevant studies have reported that histological subtypes were closely associated with metastasis.^32^ Thus, among all clinicopathologic features, we wondered whether the difference in prognosis between metastatic sites was related to the histologic subtype. Statistical differences were found in the composition of histological subtypes across metastatic sites. Thus, we focused on histological subtypes due to their statistically significant correlation and the absence of relevant studies. We plotted pie charts of the proportion of histologic subtypes in different DMS and the proportion of metastatic sites with five histologic subtypes. Based on the pie chart, ILC had the most bone metastases and IBC had the least. For IBC subtypes, patients had a higher rate of brain and lung metastasis, while IDC‐oth had the lowest proportion of brain metastasis and ILC was less likely to metastasize to lung. As for liver metastasis, IDC was more likely to metastasize to the liver while IDC‐oth and IBC were opposite. Specifically, the patients with IDC‐ILC tended to have more bone metastasis and less lung metastasis, which has been reported in TNBC.^18^ For ILC and IDC‐oth subtypes, the patients showed a higher proportion of bone metastasis and a decreased proportion of lung, liver, and brain metastasis, in accordance with published researches.^18^, ^33^ The above results showed that the distribution of histological subtypes was discrepant in four metastatic sites, suggesting that histological subtypes may have preferential distant metastasis sites (DMS), and thus lead to different prognoses of HER2+ MBC patients. Combined with the fact that patients with different metastatic sites have different outcomes, we speculated whether the prognosis of HER2+ MBC patients can be predicted based on the specific histological subtypes and specific metastatic sites. However, there were no related researches evaluating the impact of histological heterogeneity on preferential DMS and patient prognosis in HER2+ breast cancer, which aroused our further research interest.

Previous studies demonstrated that HER2+ and TNBC were associated with early relapse,^34^, ^35^ and had the shortest metastasis‐free survival and OS in comparison to patients with HR‐positive subtypes.^36^ As the distribution of histologic subtypes on distant metastasis patterns proved, we then focused on the impact on the prognosis of histological subtypes in different DMS. No statistical significance was found in most K–M prognostic survival curves, which may be due to the small sample size. However, depending on the shape of the survival curves, the prognosis of different histological subtypes of each metastatic site was different, and different metastatic sites of specific histological subtype also had different outcomes. We then explored the prognostic significance of histologic subtype in each metastasis group. Using log‐rank analysis, we further evaluated the prognosis of different histological subtypes within certain DMS. Compared with IDC, ILC in bone metastasis showed poorer DSS. We further analyzed prognosis differences of certain subtypes with various DMS. When histological subtypes were not considered, brain metastasis indicated remarkably poorest prognosis, and liver and lung metastasis also had poorer prognoses than bone metastasis. After taking histological subtypes into account, we found that the results for IDC were still consistent with the total histologic subtypes group, which has been proved in TNBC.^18^, ^37^ On the contrary, the occurrence of lung metastasis of IDC‐ILC showed the worst prognosis rather than brain metastasis, which has not been reported, indicating that the follow‐up and medical examination of patients with IDC‐ILC should pay more attention. These above analyses demonstrated that histologic subtypes predicted the prognosis of HER2+ MBC patients. Among all histological subtypes, the prognosis of ILC subtypes in bone metastasis and ILC‐IDC with lung metastasis were worth special concern.

According to HR expression status, HER2+ breast cancer was divided into two subtypes, HR+/HER2+ and HR−/HER2+, with different prognoses.^38^ We then addressed whether the role of histological subtype in determining the prognosis of patients with distant metastasis was influenced by HR expression status. The results showed that the prognosis of HR−/HER2+ subtype was generally similar as HER2+ group, while HR+/HER2+ subtype was quite different. The differences were specifically manifested in IDC‐ILC and ILC subtypes, which have not been discussed in the previous study. Based on the results of the two subgroup comparisons, we found that the prognosis of IDC‐ILC subtype with liver metastasis in HR+/HER2+ subtype as well as ILC subtype in bone metastasis of HR−/HER2+ subtype required focused monitoring.

Still, this study has some limitations. First, and inherently, retrospective studies are biased. Second, due to the small proportion of HER2+ breast cancer patients in all breast cancer, the enrolled population was limited, combined with the high heterogeneity of HER2+ breast cancer, resulting in insufficient numbers of patients in some subtypes or with certain DMS to produce significant results. The inadequacy of some subgroup data reduced the reliability of the data. In addition, limited clinical characteristics were available from SEER database, while other vital factors cannot be taken into consideration, such as differential gene expression, detailed information on anti‐HER2 treatment, symptoms associated with DMS, and the size of metastases in DMS, which indicated that more clinical data should be collected and analyzed in the future study.

## 5 CONCLUSIONS

In conclusion, our study proved the involvement of histological subtypes in determining patient outcomes in HER2+ MBC. Among all histological subtypes, the prognosis of ILC subtypes in bone metastasis and ILC‐IDC with lung metastasis deserves special attention. Furthermore, the patient prognoses of the IDC‐ILC subtype with liver metastasis of HR+/HER2+ MBC and the ILC subtype with bone metastasis of HR−/HER2+ MBC required special concerns. This study may broaden our views of the impact of histologic heterogeneity on patient prognosis and help monitor and predict the prognosis of patients with HER2+ MBC.

## AUTHOR CONTRIBUTIONS

**Yajie Wang:** Conceptualization (equal); data curation (equal); investigation (lead); project administration (equal); validation (lead); writing – original draft (lead); writing – review and editing (equal). **Yiran Liang:** Project administration (equal); writing – review and editing (equal). **Fangzhou Ye:** Investigation (equal). **Dan Luo:** Visualization (equal). **Yuhan Jin:** Formal analysis (equal). **Yaming Li:** Methodology (equal). **Wenjing Zhao:** Software (equal). **Bing Chen:** Resources (equal). **Lijuan Wang:** Validation (equal). **Qifeng Yang:** Conceptualization (equal); funding acquisition (lead); resources (equal); writing – review and editing (lead).

## FUNDING INFORMATION

This work was supported by National Key Research and Development Program (No. 2020YFA0712400), Special Foundation for Taishan Scholars (No. ts20190971), Foundation from Clinical Research Center of Shandong University (No. 2020SDUCRCA015), Qilu Hospital Clinical New Technology Developing Foundation (No. 2019‐3).

## CONFLICT OF INTEREST STATEMENT

The authors declare that the research was conducted in the absence of any commercial or financial relationships that could be construed as a potential conflict of interest.

## ETHICS STATEMENT

This article does not contain any studies with human participants or animals performed by any of the authors.

## Supporting information


Data S1.

## Data Availability

Data sharing not applicable‐no new data generated.

## References

[1] Siegel RL , Miller KD , Fuchs HE , Jemal A . Cancer statistics, 2022. CA Cancer J Clin. 2022;72(1):7‐33. 35020204 10.3322/caac.21708

[2] Fogazzi V , Kapahnke M , Cataldo A , et al. The role of MicroRNAs in HER2‐positive breast cancer: where we are and future prospective. Cancer. 2022;14(21):5326. 10.3390/cancers14215326 PMC9657949 36358746

[3] Weigelt B , Geyer FC , Reis‐Filho JS . Histological types of breast cancer: how special are they? Mol Oncol. 2010;4(3):192‐208. 20452298 10.1016/j.molonc.2010.04.004 PMC5527938

[4] Biancolella M , Testa B , Baghernajad Salehi L , D'Apice MR , Novelli G . Genetics and genomics of breast cancer: update and translational perspectives. Semin Cancer Biol. 2021;72:27‐35. 32259642 10.1016/j.semcancer.2020.03.013

[5] Wolff AC , Hammond MEH , Allison KH , et al. Human epidermal growth factor receptor 2 testing in breast cancer: American Society of Clinical Oncology/College of American Pathologists Clinical Practice Guideline Focused Update. J Clin Oncol. 2018;36(20):2105‐2122. 29846122 10.1200/JCO.2018.77.8738

[6] Cesca MG , Vian L , Cristovao‐Ferreira S , Ponde N , de Azambuja E . HER2‐positive advanced breast cancer treatment in 2020. Cancer Treat Rev. 2020;88:102033. 32534233 10.1016/j.ctrv.2020.102033

[7] Loibl S , Gianni L . HER2‐positive breast cancer. Lancet. 2017;389(10087):2415‐2429. 27939064 10.1016/S0140-6736(16)32417-5

[8] Loibl S , Poortmans P , Morrow M , Denkert C , Curigliano G . Breast cancer. Lancet. 2021;397(10286):1750‐1769. 33812473 10.1016/S0140-6736(20)32381-3

[9] Mego M , Mani SA , Cristofanilli M . Molecular mechanisms of metastasis in breast cancer—clinical applications. Nat Rev Clin Oncol. 2010;7(12):693‐701. 20956980 10.1038/nrclinonc.2010.171

[10] Bredin P , Walshe JM , Denduluri N . Systemic therapy for metastatic HER2‐positive breast cancer. Semin Oncol. 2020;47(5):259‐269. 32896428 10.1053/j.seminoncol.2020.07.008

[11] Zhang L , Li Z , Zhang J , Wu Y , Zhu Y , Tong Z . De novo metastatic breast cancer: subgroup analysis of molecular subtypes and prognosis. Oncol Lett. 2020;19(4):2884‐2894. 32218843 10.3892/ol.2020.11359 PMC7068499

[12] Weigelt B , Peterse JL , van't Veer LJ . Breast cancer metastasis: markers and models. Nat Rev Cancer. 2005;5(8):591‐602. 16056258 10.1038/nrc1670

[13] Liang Y , Zhang H , Song X , Yang Q . Metastatic heterogeneity of breast cancer: molecular mechanism and potential therapeutic targets. Semin Cancer Biol. 2020;60:14‐27. 31421262 10.1016/j.semcancer.2019.08.012

[14] Liu P , Wang Z , Ou X , et al. The FUS/circEZH2/KLF5/ feedback loop contributes to CXCR4‐induced liver metastasis of breast cancer by enhancing epithelial‐mesenchymal transition. Mol Cancer. 2022;21(1):198. 36224562 10.1186/s12943-022-01653-2 PMC9555172

[15] Azim HA , Abdel‐Malek R , Kassem L . Predicting brain metastasis in breast cancer patients: stage versus biology. Clin Breast Cancer. 2018;18(2):e187‐e195. 28888580 10.1016/j.clbc.2017.08.004

[16] Ji L , Cheng L , Zhu X , Gao Y , Fan L , Wang Z . Risk and prognostic factors of breast cancer with liver metastases. BMC Cancer. 2021;21(1):238. 33676449 10.1186/s12885-021-07968-5 PMC7937288

[17] DeSantis CE , Miller KD , Dale W , et al. Cancer statistics for adults aged 85 years and older, 2019. CA Cancer J Clin. 2019;69(6):452‐467. 31390062 10.3322/caac.21577 PMC12103238

[18] Li Y , Su P , Wang Y , et al. Impact of histotypes on preferential organ‐specific metastasis in triple‐negative breast cancer. Cancer Med. 2020;9(3):872‐881. 31814295 10.1002/cam4.2759 PMC6997059

[19] Perou CM , Sorlie T , Eisen MB , et al. Molecular portraits of human breast tumours. Nature. 2000;406(6797):747‐752. 10963602 10.1038/35021093

[20] Sorlie T , Tibshirani R , Parker J , et al. Repeated observation of breast tumor subtypes in independent gene expression data sets. Proc Natl Acad Sci U S A. 2003;100(14):8418‐8423. 12829800 10.1073/pnas.0932692100 PMC166244

[21] Jin X , Mu P . Targeting breast cancer metastasis. Breast Cancer Basic Clin Res. 2015;9(Suppl 1):23‐34. 10.4137/BCBCR.S25460 PMC4559199 26380552

[22] Zhang Y . The root cause of drug resistance in HER2‐positive breast cancer and the therapeutic approaches to overcoming the resistance. Pharmacol Ther. 2021;218:107677. 32898548 10.1016/j.pharmthera.2020.107677 PMC7855784

[23] Wang H , Zhang C , Zhang J , Kong L , Zhu H , Yu J . The prognosis analysis of different metastasis pattern in patients with different breast cancer subtypes: a SEER based study. Oncotarget. 2017;8(16):26368‐26379. 28038448 10.18632/oncotarget.14300 PMC5432264

[24] Montemagno C , Pages G . Metastatic heterogeneity of breast cancer: companion and theranostic approach in nuclear medicine. Cancer. 2020;12(4):821. 10.3390/cancers12040821 PMC7226533 32235331

[25] Smid M , Wang Y , Zhang Y , et al. Subtypes of breast cancer show preferential site of relapse. Cancer Res. 2008;68(9):3108‐3114. 18451135 10.1158/0008-5472.CAN-07-5644

[26] Zhang H , Zhu W , Biskup E , et al. Incidence, risk factors and prognostic characteristics of bone metastases and skeletal‐related events (SREs) in breast cancer patients: a systematic review of the real world data. J Bone Oncol. 2018;11:38‐50. 29511626 10.1016/j.jbo.2018.01.004 PMC5832676

[27] Kuksis M , Gao Y , Tran W , et al. The incidence of brain metastases among patients with metastatic breast cancer: a systematic review and meta‐analysis. Neuro Oncol. 2021;23(6):894‐904. 33367836 10.1093/neuonc/noaa285 PMC8168821

[28] Clark GM , Sledge GW Jr , Osborne CK , McGuire WL . Survival from first recurrence: relative importance of prognostic factors in 1,015 breast cancer patients. J Clin Oncol. 1987;5(1):55‐61. 3806159 10.1200/JCO.1987.5.1.55

[29] Hietanen P , Miettinen M , Makinen J . Survival after first recurrence in breast cancer. Eur J Cancer Clin Oncol. 1986;22(8):913‐919. 3770048 10.1016/0277-5379(86)90056-8

[30] Rudat V , El‐Sweilmeen H , Brune‐Erber I , et al. Identification of breast cancer patients with a high risk of developing brain metastases: a single‐institutional retrospective analysis. BMC Cancer. 2014;14:289. 24761771 10.1186/1471-2407-14-289 PMC4006960

[31] Wang R , Zhu Y , Liu X , Liao X , He J , Niu L . The clinicopathological features and survival outcomes of patients with different metastatic sites in stage IV breast cancer. BMC Cancer. 2019;19(1):1091. 31718602 10.1186/s12885-019-6311-z PMC6852913

[32] Rashid NS , Grible JM , Clevenger CV , Harrell JC . Breast cancer liver metastasis: current and future treatment approaches. Clin Exp Metastasis. 2021;38(3):263‐277. 33675501 10.1007/s10585-021-10080-4 PMC8211035

[33] Han B , Gu Z , Liu Z , Ling H . Clinical characteristics and survival outcomes of infiltrating lobular carcinoma: a retrospective study of 365 cases in China. Cancer Manag Res. 2022;14:647‐658. 35210861 10.2147/CMAR.S346319 PMC8858761

[34] Minicozzi P , Bella F , Toss A , et al. Relative and disease‐free survival for breast cancer in relation to subtype: a population‐based study. J Cancer Res Clin Oncol. 2013;139(9):1569‐1577. 23892409 10.1007/s00432-013-1478-1 PMC11824740

[35] van Maaren MC , de Munck L , Strobbe LJA , et al. Ten‐year recurrence rates for breast cancer subtypes in The Netherlands: a large population‐based study. Int J Cancer. 2019;144(2):263‐272. 30368776 10.1002/ijc.31914

[36] Hoeferlin LA , Ec C , Park MA . Challenges in the treatment of triple negative and HER2‐overexpressing breast cancer. J Surg Sci. 2013;1(1):3‐7. 24818173 PMC4012677

[37] Tseng LM , Hsu NC , Chen SC , et al. Distant metastasis in triple‐negative breast cancer. Neoplasma. 2013;60(3):290‐294. 23373998 10.4149/neo_2013_038

[38] Goldhirsch A , Wood WC , Coates AS , et al. Strategies for subtypes—dealing with the diversity of breast cancer: highlights of the St. Gallen International Expert Consensus on the Primary Therapy of Early Breast Cancer. Ann Oncol. 2011;22(8):1736‐1747. 21709140 10.1093/annonc/mdr304 PMC3144634

