# Supplementary material for: Histologic heterogeneity predicts patient prognosis of HER2‐positive metastatic breast cancer: A retrospective study based on SEER database
Source: Cancer Med. 2023 Aug 21;12(18):18597–610. doi: 10.1002/cam4.6469 (PMC10557902; doi:10.1002/cam4.6469)
Supplement: Supplementary file 1 — Data S1. [file CAM4-12-18597-s001.docx]

*Supplementary Material*

Histologic heterogeneity predicts patient prognosis of HER2-positive metastatic breast cancer: A retrospective study based on SEER database

Yajie Wang^1^, Yiran Liang^1^, Fangzhou Ye^1^, Dan Luo^1^, Yuhan Jin^1^, Yaming Li^1^, Wenjing Zhao^2^, Bing Chen^2^, Lijuan Wang^2^, and Qifeng Yang^1,2,3,*^

^1^Department of Breast Surgery, General Surgery, Qilu Hospital of Shandong University, Jinan, Shandong, 250012, China

^2^Pathology Tissue Bank, Qilu Hospital of Shandong University, Jinan, Shandong, 250012, China

^3^Research Institute of Breast Cancer, Shandong University, Jinan, Shandong, 250012, China

*** Correspondence:**Qifeng Yang
qifengy_sdu@163.com

**SUPPLEMENTARY TABLE 1** Distribution and differences of histological types in each metastasis type

| **Characteristics** | **Bone metastasis** | **Brain metastasis** | **Liver metastasis** | **Lung metastasis** |
| --- | --- | --- | --- | --- |
| **Histologic subtype** |  |  |  | **P = .001** |
| IDC | 505 (84.5) | 26 (83.9) | 279 (91.5) | 221 (92.1) |
| ILC | 36 (6.0) | 1 (3.2) | 6 (2.0) | 1 (0.4) |
| IDC-ILC | 29 (4.9) | 2 (6.5) | 13 (4.3) | 6 (2.5) |
| IDC-oth | 13 (2.2) | 0 (0) | 3 (1.0) | 1 (0.4) |
| IBC | 15 (2.5) | 2 (6.5) | 4 (1.3) | 11 (4.6) |

**SUPPLEMENTARY TABLE 2** Logistic univariate analysis of risk factors for different distant metastasis

|  | **Bone Metastasis** | | **Brain Metastasis** | | | **Liver Metastasis** | | **Lung Metastasis** | | | **Total single metastasis** | |
| --- | --- | --- | --- | --- | --- | --- | --- | --- | --- | --- | --- | --- |
|  | **OR (95% CI)** | **P** | **OR (95% CI)** | **P** | **OR (95% CI)** | | **P** | | **OR (95% CI)** | **P** | **OR (95% CI)** | **P** |
| **Patient age, years** |  |  |  |  |  | |  | |  |  |  |  |
| ＜50 | REF^3^ | REF | REF | REF | REF | | REF | | REF | REF | REF | REF |
| 50-75 | 0.967(0.811-1.153) | .710 | 1.199(0.549-2.619) | .649 | 0.796(0.627-1.010) | | .060 | | **1.513(1.107-2.068)** | **.009** | 0.999(0.879-1.135) | .984 |
| ＞75 | 0.823(0.575-1.179) | .288 | 0.495(0.063-3.913) | .505 | 0.671(0.402-1.120) | | .127 | | **2.608(1.670-4.074)** | **＜.001** | 1.024(0.805-1.303) | .845 |
| **Race** |  |  |  |  |  | |  | |  |  |  |  |
| White | REF | REF | REF | REF | REF | | REF | | REF | REF | REF | REF |
| Black | **1.431(1.155-1.773)** | **.001** | **2.364(1.040-5.371)** | **.040** | **1.374(1.017-1.856)** | | **.039** | | 1.364(0.973-1.911) | .071 | **1.423(1.219-1.662)** | **＜.001** |
| Other^1^ | **0.648(0.476-0.883)** | **.006** | 0.955(0.284-3.217) | .941 | 0.670(0.439-1.023) | | .063 | | **0.560(0.335-0.935)** | **.027** | **0.643(0.515-0.803)** | **＜.001** |
| Unknown | 0.830(0.307-2.241) | .713 | —^4^ | — | — | | — | | — | — | 0.421(0.156-1.133) | .087 |
| **Marital status** |  |  |  |  |  | |  | |  |  |  |  |
| Unmarried^2^ | REF | REF | REF | REF | REF | | REF | | REF | REF | REF | REF |
| Married | **0.627(0.531-0.742)** | **＜.001** | **0.454(0.222-0.927)** | **.019** | **0.726(0.575-0.916)** | | **.007** | | **0.534(0.410-0.695)** | **＜.001** | **0.624(0.553-0.704)** | **＜.001** |
| Unknown | 1.014(0.719-1.429) | .938 | 0.415(0.055-3.111) | .392 | 0.892(0.529-1.502) | | .667 | | 0.988(0.585-1.669) | .988 | 0.959(0.744-1.236) | .746 |
| **Grade** |  |  |  |  |  | |  | |  |  |  |  |
| Well/Moderately;GradeⅠ/Ⅱ | REF | REF | REF | REF | REF | | REF | | REF | REF | REF | REF |
| Poorly/Undifferentiated;GradeⅢ/Ⅳ | **0.789(0.663-0.939)** | **.007** | 2.591(0.967-6.941) | .058 | 1.278(0.997-1.640) | | .053 | | **2.161(1.585-2.946)** | **＜.001** | 1.114(0.980-1.265) | .098 |
| Unknown | 2.598(1.994-3.383) | ＜.001 | 11.580(3.670-36.538) | ＜.001 | 2.499(1.643-3.800) | | ＜.001 | | 2.965(1.750-5.024) | ＜.001 | 2.736(2.227-3.362) | ＜.001 |
| **Laterality** |  |  |  |  |  | |  | |  |  |  |  |
| Left | REF | REF | REF | REF | REF | | REF | | REF | REF | REF | REF |
| Right | 0.888(0.754-1.045) | .153 | 0.869(0.428-1.764) | .698 | 1.069(0.853-1.341) | | .562 | | **1.292(1.000-1.670)** | **.050** | 1.006(0.894-1.131) | .924 |
| Bilateral | **34.086(13.728-105.661)** | **＜.001** | — | — | **11.566(1.438-93.048)** | | **.021** | | **32.645(6.852-155.525)** | **＜.001** | **29.304(11.524-74.516)** | **＜.001** |
| Unknown | — | — | — | — | — | | — | | — | — | — | — |
| **Histology** |  |  |  |  |  | |  | |  |  |  |  |
| IDC | REF | REF | REF | REF | REF | | REF | | REF | REF | REF | REF |
| ILC | **2.362(1.671-3.338)** | **＜.001** | 1.274(0.173-9.403) | .812 | 0.712(0.316-1.605) | | .413 | | 0.150(0.021-1.070) | .058 | **1.414(1.037-1.929)** | **.029** |
| IDC-ILC | **1.608(1.099-2.351)** | **.014** | 2.154(0.510-9.088) | .296 | 1.305(0.745-2.284) | | .352 | | 0.760(0.337-1.715) | .509 | **1.358(1.014-1.818)** | **.040** |
| IDC-oth | 0.899(0.516-1.566) | .706 | — | — | 0.375(0.120-1.174) | | .092 | | 0.158(0.022-1.128) | .066 | **0.576(0.354-0.935)** | **.026** |
| IBC | **5.445(3.171-9.350)** | **＜.001** | **14.102(3.314-60.006)** | **＜.001** | 2.628(0.966-7.154) | | .059 | | **9.125(4.866-17.110)** | **＜.001** | **5.690(3.851-8.407)** | **＜.001** |
| **Tumor size(mm)** |  |  |  |  |  | |  | |  |  |  |  |
| ≤50 | REF | REF | REF | REF | REF | | REF | | REF | REF | REF | REF |
| ＞50 | **7.018(5.907-8.338)** | **＜.001** | **5.920(2.815-12.452)** | **＜.001** | **5.699(4.482-7.245)** | | **＜.001** | | **9.790(7.514-12.754)** | **＜.001** | **7.124(6.288-8.072)** | **＜.001** |
| Unknown | 10.225(7.577-13.799) | ＜.001 | 12.822(4.240-38.772) | ＜.001 | 9.579(6.408-14.321) | | ＜.001 | | 4.538(2.277-9.042) | ＜.001 | 9.106(7.221-11.482) | ＜.001 |
| **Node stage** |  |  |  |  |  | |  | |  |  |  |  |
| Negative | REF | REF | REF | REF | REF | | REF | | REF | REF | REF | REF |
| Positve | **5.110(4.182-6.244)** | **＜.001** | **6.819(2.601-17.877)** | **＜.001** | **5.119(3.868-6.774)** | | **＜.001** | | **7.290(5.145-10.328)** | **＜.001** | **5.509(4.757-6.382)** | **＜.001** |
| Unknown | 26.592(17.063-41.442) | ＜.001 | 47.105(9.060-244.920) | ＜.001 | 28.039(15.573-50.482) | | ＜.001 | | 21.693(9.519-49.440) | ＜.001 | 26.625(18.849-37.607) | ＜.001 |
| **Radiotherapy status** |  |  |  |  |  | |  | |  |  |  |  |
| No/Unknown | REF | REF | REF | REF | REF | | REF | | REF | REF | REF | REF |
| Yes | **0.393(0.326-0.472)** | **＜.001** | 1.052(0.520-2.129) | .888 | **0.231(0.171-0.311)** | | **＜.001** | | **0.119(0.077-0.183)** | **＜.001** | **0.296(0.257-0.341)** | **＜.001** |
| **Chemotherapy status** |  |  |  |  |  | |  | |  |  |  |  |
| No/Unknown | REF | REF | REF | REF | REF | | REF | | REF | REF | REF | REF |
| Yes | **1.302(1.065-1.593)** | **.010** | 0.808(0.372-1.755) | .590 | **1.438(1.077-1.920)** | | **.014** | | 1.138(0.840-1.543) | .405 | **1.279(1.107-1.478)** | **.001** |

1 Other Includes American Indian/Alaskan native, Asian/Pacific Islander, and others—unspecified.

2 Unmarried means divorced, separated, single (never married), unmarried, domestic partner, and widowed.

3 For calculation of OR value, a group of patients were defined as reference.

4 The number of patients was not enough for further calculation.

**SUPPLEMENTARY TABLE 3** Logistic multivariate analysis of risk factors for different distant metastasis

|  | **Bone Metastasis** | | | **Brain Metastasis** | | | **Liver Metastasis** | | | **Lung Metastasis** | | **Total single metastasis** | | |
| --- | --- | --- | --- | --- | --- | --- | --- | --- | --- | --- | --- | --- | --- | --- |
|  | **OR (95% CI)** | **P** | **OR (95% CI)** | | **P** | **OR (95% CI)** | | **P** | **OR (95% CI)** | | **P** | | **OR (95% CI)** | **P** |
| **Patient age, years** |  |  |  | |  |  | |  |  | |  | |  |  |
| ＜50 | —^3^ | — | — | | — | — | | — | REF^4^ | | REF | | — | — |
| 50-75 | — | — | — | | — | — | | — | **1.711(1.242-2.358)** | | **.001** | | — | — |
| ＞75 | — | — | — | | — | — | | — | **2.256(1.414-3.601)** | | **.001** | | — | — |
| **Race** |  |  |  | |  |  | |  |  | |  | |  |  |
| White | REF | REF | REF | | REF | REF | | REF | REF | | REF | | REF | REF |
| Black | 1.206(0.962-1.513) | .105 | 1.874(0.804-4.367) | | .146 | 1.181(0.863-1.616) | | .298 | 1.062(0.774-1.515) | | .742 | | **1.190(1.006-1.408)** | **.042** |
| Other^1^ | **0.652(0.476-0.895)** | **.008** | 1.057(0.312-3.582) | | .929 | **0.624(0.407-0.957)** | | **.031** | **0.521(0.309-0.878)** | | **.014** | | **0.622(0.493-0.784)** | **＜.001** |
| Unknown | 0.436(0.153-1.245) | .121 | — | | — | — | | — | — | | — | | 0.205(0.073-0.578) | .003 |
| **Marital status** |  |  |  | |  |  | |  |  | |  | |  |  |
| Unmarried^2^ | REF | REF | REF | | REF | REF | | REF | REF | | REF | | REF | REF |
| Married | **0.777(0.651-0.928)** | **.005** | 0.536(0.252-1.140) | | .105 | 0.915(0.718-1.166) | | .473 | **0.744(0.562-0.984)** | | **.038** | | **0.803(0.705-0.915)** | **.001** |
| Unknown | 1.005(0.697-1.449) | .980 | 0.424(0.056-3.226) | | .408 | 0.946(0.554-1.613) | | .837 | 1.154(0.668-1.992) | | .608 | | 1.028(0.782-1.351) | .844 |
| **Grade** |  |  |  | |  |  | |  |  | |  | |  |  |
| Well/Moderately;GradeⅠ/Ⅱ | REF | REF | — | | — | — | | — | REF | | REF | | — | — |
| Poorly/Undifferentiated;GradeⅢ/Ⅳ | **0.653(0.545-0.783)** | **＜.001** | — | | — | — | | — | **1.556(1.131-2.140)** | | **.007** | | — | — |
| Unknown | 1.530(1.146-2.043) | .004 | — | | — | — | | — | 1.705(0.973-2.988) | | .062 | | — | — |
| **Laterality** |  |  |  | |  |  | |  |  | |  | |  |  |
| Left | REF | REF | — | | — | REF | | REF | REF | | REF | | REF | REF |
| Right | 0.887(0.749-1.050) | .165 | — | | — | 1.053(0.837-1.326) | | .657 | 1.298(0.997-1.690) | | .053 | | 0.999(0.883-1.131) | .990 |
| Bilateral | **6.044(1.745-20.934)** | **.005** | — | | — | 1.694(0.171-16.773) | | .652 | **9.283(1.396-61.719)** | | **.021** | | **6.416(2.055-20.030)** | **.001** |
| Unknown | — | — | — | | — | — | | — | — | | — | | — | — |
| **Histology** |  |  |  | |  |  | |  |  | |  | |  |  |
| IDC | REF | REF | REF | | REF | REF | | REF | REF | | REF | | REF | REF |
| ILC | **1.583(1.085-2.310)** | **.017** | 1.105(0.146-8.340) | | .923 | 0.551(0.239-1.272) | | .163 | **0.097(0.013-0.724)** | | **.023** | | 1.146(0.817-1.608) | .430 |
| IDC-ILC | 1.439(0.970-2.135) | .071 | 2.060(0.481-8.823) | | .330 | 1.259(0.711-2.231) | | .429 | 0.782(0.341-1.794) | | .562 | | 1.335(0.981-1.816) | .066 |
| IDC-oth | 0.882(0.498-1.562) | .667 | — | | — | 0.376(0.119-1.186) | | .095 | 0.169(0.023-1.216) | | .077 | | **0.587(0.355-0.969)** | **.037** |
| IBC | 1.141(0.644-2.020) | .652 | 4.453(0.966-20.520) | | .055 | 0.676(0.243-1.880) | | .453 | 1.758(0.891-3.469) | | .104 | | 1.336(0.880-2.029) | .173 |
| **Tumor size(mm)** |  |  |  | |  |  | |  |  | |  | |  |  |
| ≤50 | REF | REF | REF | | REF | REF | | REF | REF | | REF | | REF | REF |
| ＞50 | **4.445(3.683-5.370)** | **＜.001** | **3.152(1.388-7.162)** | | **.006** | **3.809(2.948-4.922)** | | **＜.001** | **6.001(4.504-7.996)** | | **＜.001** | | **4.524(3.947-5.185)** | **＜.001** |
| Unknown | 5.363(3.815-7.539) | ＜.001 | 8.431(2.386-29.790) | | .001 | 5.825(3.722-9.119) | | ＜.001 | 2.381(1.128-5.025) | | .023 | | 5.493(4.233-7.127) | ＜.001 |
| **Node stage** |  |  |  | |  |  | |  |  | |  | |  |  |
| Negative | REF | REF | REF | | REF | REF | | REF | REF | | REF | | REF | REF |
| Positve | **3.650(2.937-4.535)** | **＜.001** | **4.508(1.654-12.285)** | | **.003** | **3.815(2.832-5.140)** | | **＜.001** | **4.688(3.244-6.777)** | | **＜.001** | | **3.894(3.324-4.562)** | **＜.001** |
| Unknown | 7.008(4.207-11.674) | ＜.001 | 13.153(2.075-83.351) | | .006 | 8.788(4.529-17.052) | | ＜.001 | 7.261(2.963-17.795) | | ＜.001 | | 8.021(5.416-11.879) | ＜.001 |
| **Radiotherapy status** |  |  |  | |  |  | |  |  | |  | |  |  |
| No/Unknown | REF | REF | — | | — | REF | | REF | REF | | REF | | REF | REF |
| Yes | **0.348(0.287-0.422)** | **＜.001** | — | | — | **0.193(0.142-0.261)** | | **＜.001** | **0.101(0.065-0.156)** | | **＜.001** | | **0.254(0.219-0.296)** | **＜.001** |
| **Chemotherapy status** |  |  |  | |  |  | |  |  | |  | |  |  |
| No/Unknown | REF | REF | — | | — | REF | | REF | — | | — | | REF | REF |
| Yes | 1.146(0.917-1.431) | .230 | — | | — | 1.336(0.982-1.819) | | .065 | — | | — | | 1.128(0.960-1.324) | .143 |

1 Other Includes American Indian/Alaskan native, Asian/Pacific Islander, and others—unspecified.

2 Unmarried means divorced, separated, single (never married), unmarried, domestic partner, and widowed.

3 The number of patients was not enough for further calculation.

4 For calculation of OR value, a group of patients were defined as reference.

**SUPPLEMENTARY FIGURES**


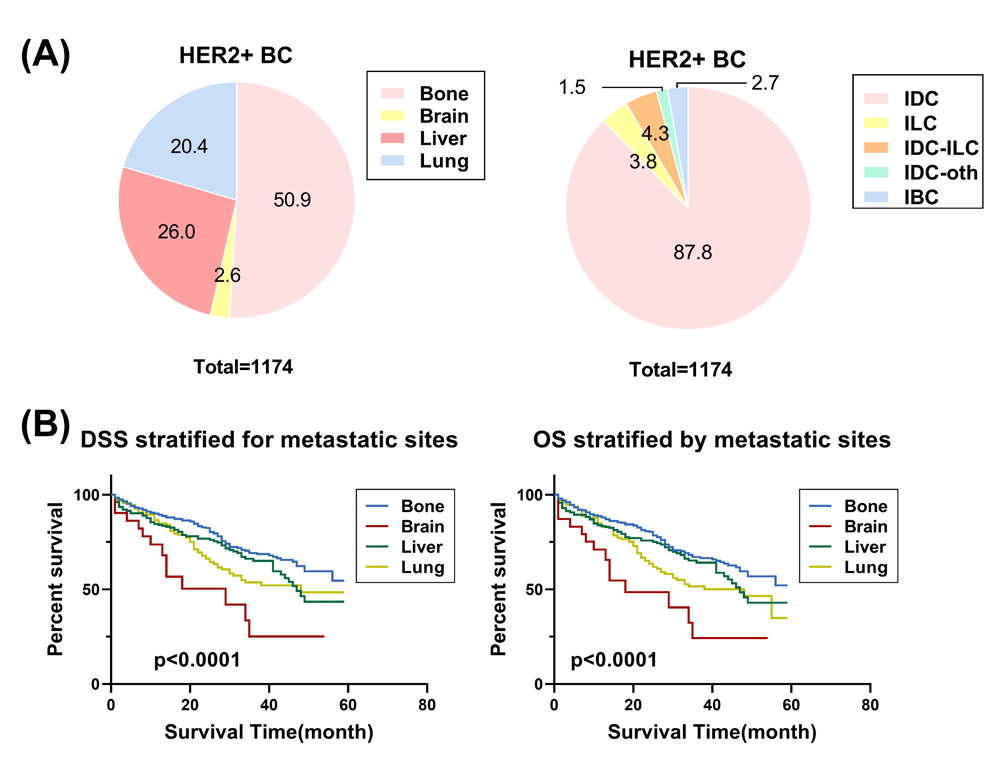


**SUPPLEMENTARY FIGURE 1** The proportion and prognosis of different metastatic sites in HER2+ MBC. (A) The proportion of different metastatic sites and histologic subtypes in HER2+ MBC. (B) The prognosis of different metastatic sites in HER2+ MBC


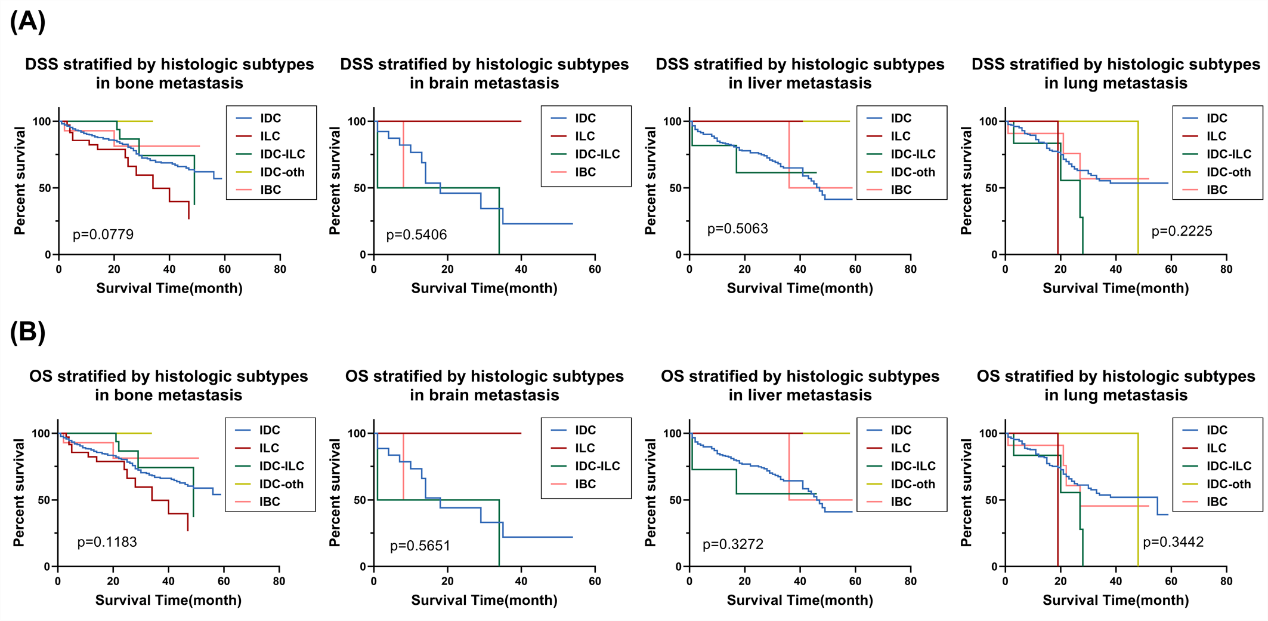


**SUPPLEMENTARY FIGURE 2** Kalan-Meier survival curve of DSS and OS stratified by histologic subtypes in specific distant metastatic site. (A) DSS stratified by histologic subtypes in specific distant metastatic site. (B) OS stratified by histologic subtypes in specific distant metastatic site


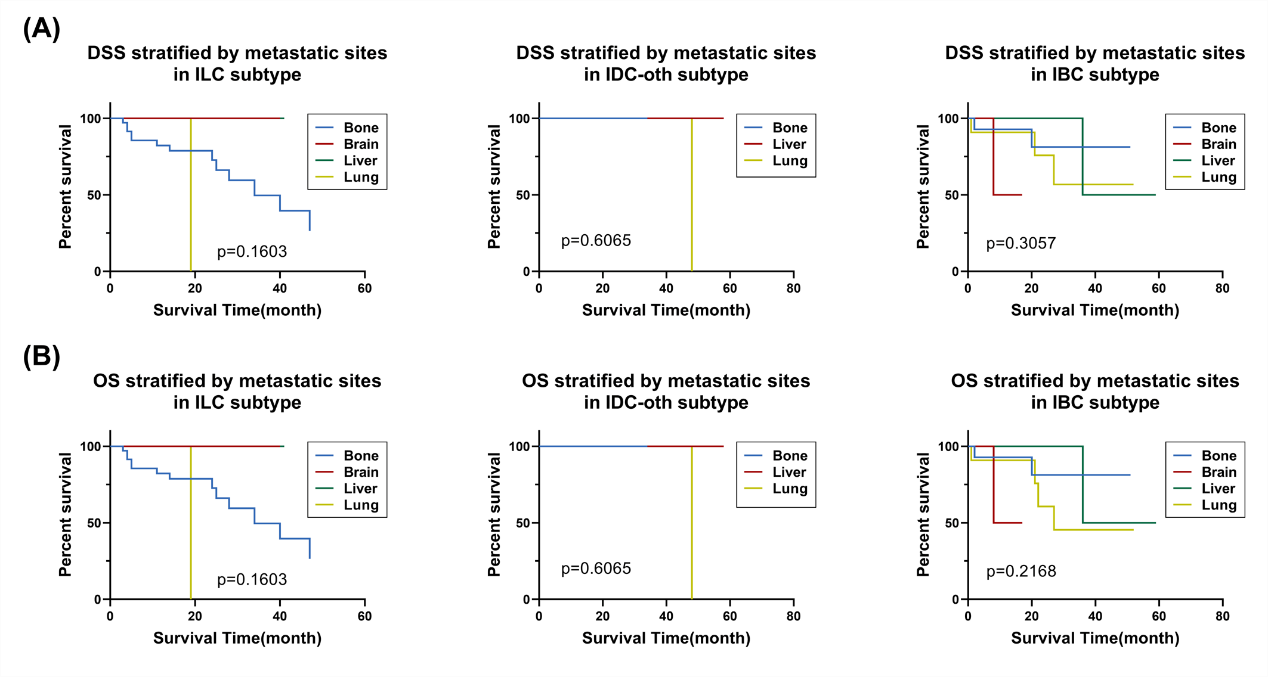


**SUPPLEMENTARY FIGURE 3** Kalan-Meier survival curve of DSS and OS stratified by metastatic sites in ILC, IDC-oth, and IBC subtypes. (A) DSS stratified by metastatic sites in ILC, IDC-oth, and IBC subtypes. (B) OS stratified by metastatic sites in ILC, IDC-oth, and IBC subtypes
